# CD8^+^ T Cell-Associated Gene Signature Correlates With Prognosis Risk and Immunotherapy Response in Patients With Lung Adenocarcinoma

**DOI:** 10.3389/fimmu.2022.806877

**Published:** 2022-02-22

**Authors:** Minghui Zhang, Jianli Ma, Qiuyue Guo, Shuang Ding, Yan Wang, Haihong Pu

**Affiliations:** ^1^ Department of Medical Oncology, Harbin Medical University Cancer Hospital, Harbin, China; ^2^ Clinical Trial Center, Harbin Medical University Cancer Hospital, Harbin, China; ^3^ Department of Radiation Oncology, Harbin Medical University Cancer Hospital, Harbin, China

**Keywords:** lung adenocarcinoma, CD8^+^ T lymphocytes, gene signature, prognosis, immunotherapy response

## Abstract

The presence of infiltrating CD8^+^ T lymphocytes in the tumor microenvironment of lung adenocarcinoma (LUAD) is correlated with improved patient prognosis, but underlying regulatory mechanisms remain unknown. To identify biomarkers to improve early diagnosis and treatment of LUAD, we downloaded 13 immune cell line-associated datasets from the GEO database. We identified CD8^+^ T cell-associated genes *via* weighted correlation network analysis. We constructed molecular subtypes based on CD8^+^ T cell-associated genes and constructed a multi-gene signature. We identified 252 CD8^+^ T cell-associated genes significantly enriched in immune function-related pathways and two molecular subtypes of LUAD (immune cluster 1 [IC1] and IC2) using our CD8^+^ T cell-associated gene signature. Patients with the IC2 subtype had a higher tumor mutation burden and lower immune infiltration scores, whereas those with the IC1 subtype were more sensitive to immune checkpoint inhibitors. Prioritizing the top candidate genes to construct a 10-gene signature, we validated our model using independent GSE and TCGA datasets to confirm its robustness and stable prognostic ability. Our risk model demonstrated good predictive efficacy using the Imvigor210 immunotherapy dataset. Thus, we established a novel and robust CD8^+^ T cell-associated gene signature, which could help assess prognostic risk and immunotherapy response in LUAD patients.

## 1 Introduction

Lung cancer, the leading cause of cancer-related deaths worldwide (1, 2), is distinguished by two histological subtypes: small cell lung cancer and non-small cell lung cancer. The most common subtype of non-small cell lung cancer is lung adenocarcinoma (LUAD), which accounts for 40% of lung cancer cases. LUAD incidence has increased in recent decades, posing a significant danger to human health and life. Thus, early diagnosis and treatment of LUAD have become essential research aims (3, 4). Early diagnosis of LUAD is challenging because of the lack of early biomarkers and symptoms. Consequentially, local progression likely ensues by the time patients exhibit symptoms and receive a differential diagnosis, missing the optimal time for surgical treatment (5–7). Exploring prognostic methods specific to LUAD patients is urgently needed to provide personalized treatment and management plans.

Imbalance in the immune tumor microenvironment (TME) is one of the most conspicuous features of tumors (8). The TME contains various cell types, including tumor cells, stromal cells (epithelial cells, fibroblasts, and adipocytes), and immune cells (T cells, B cells, and macrophages) (9). Among them, the adaptive immune responses mediated by immune cells play a critical role in tumor progression. Particularly, CD8^+^ T cells are the predominant antitumor effector cells in the TME that mainly play a cytotoxic role, but their function is impaired by the presence of various immunosuppressive cells or molecules in the TME (10). Additionally, co-inhibitory molecules on the surface of CD8^+^ T cells are upregulated as the immune response dampens, such as programmed death protein 1 (PD-1), and the expression of cytotoxic T lymphocyte-associated protein 4 (CTLA4) increases (11, 12). These co-inhibitory molecules bind to ligands in the TME, ultimately leading to T cell exhaustion (13). Therefore, elucidating the regulatory mechanisms associated with CD8^+^ T cells in the TME is critical.

Previous studies have demonstrated that infiltration of CD8^+^ T lymphocytes in LUAD tissues results in significant improvement in patient prognosis (14). However, these CD8^+^ T lymphocytes are often dysfunctional and fail to initiate an immune response to eliminate tumor cells (15). For example, IL-38 reportedly promotes LUAD proliferation by inhibiting the number of CD8^+^ T lymphocytes in the TME (16). Conversely, the aspartic acid in the TME promotes CD8^+^ T cell activation through the LCK signaling pathway and exerts antitumor effects (17). These findings suggest that targeting and regulating the function of CD8^+^ T cells has potential clinical implications. Indeed, CD8^+^ T cell infiltration density combined with TNM stage is an independent predictor of poor prognosis in LUAD patients (18). However, the regulatory mechanisms and clinical significance associated with CD8^+^ T cells have not been fully elucidated in LUAD.

The goal of this study, through the use of immune cell line-associated datasets, was to identify CD8^+^ T cell-associated marker genes to construct a multi-gene signature and develop a prognostic risk model for LUAD. The study findings will help improve predictions of prognosis and immunotherapeutic response in LUAD patients, thus enhancing treatment outcomes.

## 2 Materials and Methods

### 2.1 Data Sources

Thirteen immune cell line-associated datasets were downloaded from the Gene Expression Omnibus: GSE13906, GSE23371, GSE27291, GSE27838, GSE28490, GSE28726, GSE37750, GSE39889, GSE42058, GSE49910, GSE59327, GSE6863, and GSE8059. The datasets included microarray expression data for 14 immune cell types, including B cells, CD4^+^ T cells, CD8^+^ T cells, dendritic cells, eosinophils, gamma-delta T cells, immature dendritic cells, lymphocytes, monocytes, myeloid dendritic cells, natural killer cells, neutrophils, plasmacytoid dendritic cells, and natural killer T cells. RNA-sequencing data and clinical information from samples in the TCGA-LUAD project were downloaded using the National Cancer Institute Genomic Data Commons Application Programming Interface.

In addition, microarray and time to live data were downloaded from the Gene Expression Omnibus for datasets GSE37745, GSE19188, GSE50081, GSE30219, and GSE31210.

### 2.2 Data Pre-Processing

Each immune cell line-associated dataset was processed using the RMA algorithm of the *affy* package in R software. Batch effect corrections were performed using the “removeBatchEffect’ function of the *limma* package in R software. The probes were subsequently converted to gene symbols based on the annotation file.

The following steps were additionally performed on the RNA-Seq data of samples from the TCGA-LUAD dataset. Samples without clinical follow-up information or living status were removed. The ensemble gene IDs were then converted to gene symbols. The median value was taken when multiple gene symbols were expressed across cell lines. Additionally, samples were filtered out if > 50% gene expression was less than 1.

The following steps were additionally performed on sample data from the GSE-LUAD dataset. Normal tissue samples and samples without clinical follow-up information, overall survival data, or living status were removed. The probes were then converted to gene symbols based on the annotation file. Principal component analysis was performed to verify the batch effect correction for the expression profiles of the GSE dataset (**Supplementary Figure 1**), revealing no significant differences between the different datasets following batch effect correction.

After pre-processing and quality control steps, the final dataset consisted of 500 samples from the TCGA-LUAD cohort and 582 samples from the GSE-LUAD cohort.

### 2.3 Identification of CD8^+^ T Cell Marker Genes

#### 2.3.1 Pre-Processing of Immune Cell Data

The 13 immune cell line-associated datasets were merged, and a batch effect correction was performed as previously described. Principal component analysis was performed to verify the batch effect correction for the expression profiles of the immune cell dataset (**Supplementary Figure 2**). Our analysis indicated that the different datasets were dispersed before but harmonized after the batch effect correction.

### 2.4 Weighted Correlation Network Analysis-Based Co-Expression Analysis of CD8^+^ T Cell-Associated Genes

The 179 expression profiles from the immune cell dataset were clustered using hierarchical clustering, and distances between genes were calculated using Pearson’s correlation coefficient. Furthermore, a weighted co-expression network was constructed using the *WGCNA* package in R software. We found that the co-expression network was scale-free, i.e., log(k) of the node with connectivity k was negatively correlated with log(P(k)) of the probability P of occurrence of that node, and the correlation coefficient > 0.85. The expression matrix was converted into an adjacency matrix and subsequently into a topology matrix. The genes were clustered using the average linkage hierarchical clustering method based on the topological overlap matrix. The minimum number of genes per gene-network module was set to 100, according to the criteria of the hybrid dynamic tree cut. The eigenvector values of each module were calculated after determining the gene modules using the *dynamicTreecut* package in R software. The modules were clustered and merged with closer modules to form new modules with the following settings: applied height = 0.25, deepSplit = 2, and minModuleSize = 100.

Further, Kyoto Encyclopedia of Genes and Genomes (KEGG) pathway analysis and Gene Ontology functional enrichment analysis of the CD8^+^ T cell-associated genes were performed using the *clusterProfiler* package in R software (v3.14.0).

### 2.5 Molecular Subtyping Based on CD8^+^ T Cell-Associated Genes

Univariate cox analysis of CD8 T cells genes was performed from the TCGA and GEO cohorts and obtain their intersection genes. Subsequently, cluster analysis was performed using common CD8 T cells prognostic genes. Specifically, the 500 TCGA-LUAD samples were clustered using the *ConsensusClusterPlus* package in R software based on the expression of CD8 T cells prognostic genes. The optimal number of clusters was determined according to the cumulative distribution function (CDF). The CDF delta area curve value was used to select the optimal number of clusters with the greatest stability. Subsequently, the immune subtype features of different clusters were analyzed. The same analysis was performed on the GEO-LUAD cohort to demonstrate the ability to distinguish molecular subtypes across different study cohorts.

## 3 Results

### 3.1 Selection and Analysis of CD8^+^ T Cell-Associated Genes

**Figure 1:**
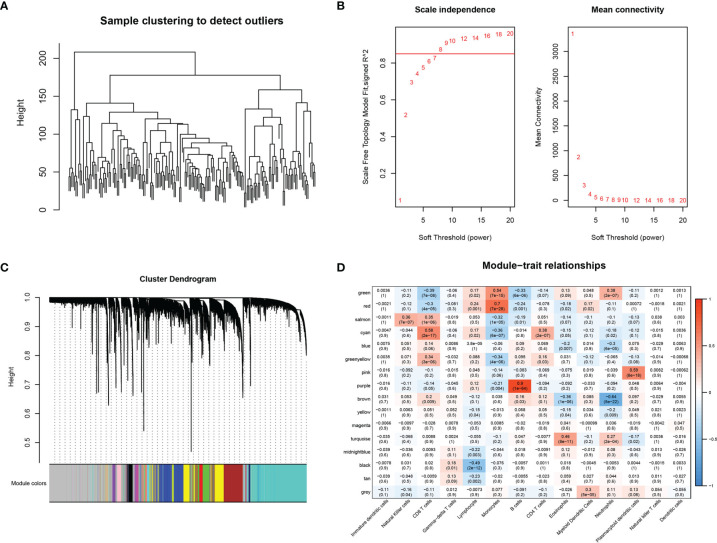
WGCNA-based co-expression analysis of CD8^+^ T cell-associated genes. **(A)** Sample clustering analysis. **(B)** Analysis of network topology for various soft-thresholding powers. **(C)** Gene dendrogram and module colors. **(D)** Correlation results between the 16 modules and each clinical phenotype.

The 179 expression profiles in the immune cell line-associated dataset were clustered using hierarchical clustering, as shown in **Figure 1A**. To ensure the network was scale-free, β = 8 was chosen (**Figure 1B**). A total of 16 modules were obtained (**Figure 1C**), from which X modules were categorized as gray, where the gene set could not be aggregated into other modules. The correlations between modules and immune cells were further analyzed (**Figure 1D**), indicating that the cyan-colored module had the most significant positive correlation with CD8^+^ T cells, was less correlated with other immune cells, and contained 252 genes.

**Figure 2:**
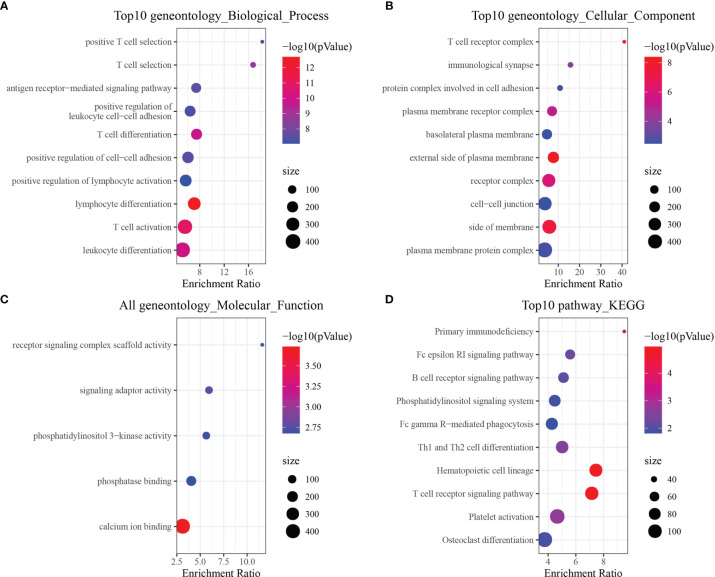
Functional enrichment analysis of CD8^+^ T cell-associated genes. **(A)** Biological process annotation map of genes in the cyan-colored module. **(B)** Molecular function annotation map of genes in the cyan-colored module. **(C)** Cellular component annotation map of genes in the cyan-colored module. **(D)** Kyoto Encyclopedia of Genes and Genomes annotation map of genes in the cyan-colored module.

Gene Ontology enrichment analysis was performed to determine which signaling pathways were enriched in the CD8^+^ T cell module, indicating that 129 genes were significantly enriched in biological processes (P < 0.05), of which the top ten biological process terms are shown in **Figure 2A**. Twelve genes were significantly enriched in pathways related to cellular component (P < 0.05), of which the top 10 cellular component pathways are shown in **Figure 2B**. Five genes were significantly enriched in pathways related to molecular function (P < 0.05) (**Figure 2C**). We also identified 19 pathways that were significantly enriched *via* KEGG pathway enrichment analysis (P < 0.05), of which the top 10 annotations are shown in **Figure 2D**. The annotation results indicated that these genes were closely associated with immune function and pathways.

### 3.2 Molecular Subtyping Based on CD8^+^ T Cell-Associated Genes

**Figure 3:**
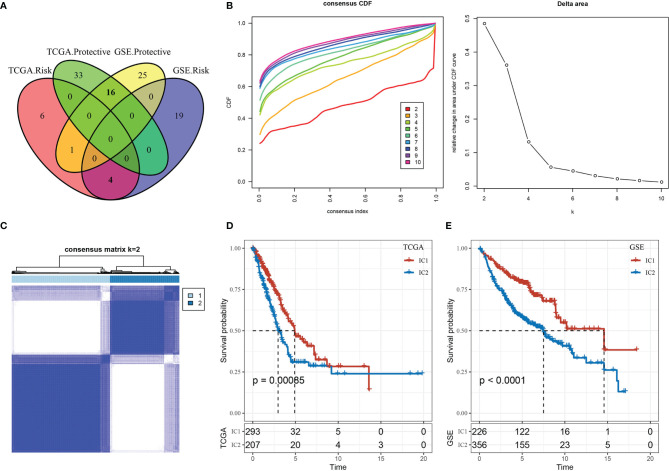
Immune clusters (ICs) in lung adenocarcinoma (LUAD). **(A)** Venn diagram displaying the intersection of CD8^+^ T cell genes significantly associated with prognosis between the two cohorts (TCGA-LUAD and GSE-LUAD). **(B)** Cumulative distribution function (CDF) curve and CDF delta area curve of TCGA-LUAD samples: delta area curve of consensus clustering indicates the relative change in area under the CDF curve for each category number k compared with k–1, where the horizontal axis represents the category number k, and the vertical axis represents the relative change in area under CDF curve. **(C)** Heat map of sample clustering at consensus k = 2. **(D)** Survival curves for the molecular subtypes in the TCGA-LUAD cohort. **(E)** Survival curves for the molecular subtypes in the GSE-LUAD cohort.

Univariate analysis was performed on the TCGA-LUAG and GSE-LUAG datasets to identify whether tumor-infiltrating CD8^+^ T cells were associated with LUAD patient outcomes. Sixty and 65 genes were associated with prognosis in the TCGA-LUAD and GSE-LUAD datasets, respectively. Among these, only 20 genes were present in both datasets, as shown in **Figure 3A**, suggesting that the expression of CD8^+^ T cell-associated genes may be inconsistent across datasets obtained using different sequencing platforms, as well as across cohorts. 20 CD8^+^ T cell-associated genes include AGMAT, AMIGO1, AQP3, ATP8B2, BEX5, CD69, DVL3, EPHX2, GPRASP1, HEMGN, IL7R, LRRN3, MAL, MGP, NR3C2, PPP1R13B, SMAGP, STRN4, TCEA3 and ZNF540.We subsequently proceeded with downstream analysis using the 20 CD8^+^ T cell-associated genes that were significantly associated with prognosis (P < 0.05).We further analyzed the expression of 20 genes in tumor tissues and normal tissues, and found that the expression of 20 genes in most tumors was significantly different from that in normal tissues (**Supplementary Figure 3**). 345 pathways and corresponding genes were obtained from KEGG, and then used 20 genes to match pathway, of which 7 genes can be matched. Then, Cytoscape software was used to map pathways and genes interactively, as shown in the **Supplementary Figure 4**. We obtained the original data of single-cell data GSE148071 (42 samples in total), retained 18 patient samples of lung adenocarcinoma, and performed cell annotation analysis. The results are shown that most genes are significantly expressed in CD8+ T cells (**Supplementary Figure 5**).

Performing a consensus clustering analysis of the TCGA-LUAD cohort, we obtained two stable immune clusters (ICs) by choosing k = 2, as shown by the CDF delta area curve associated with molecular subtypes (**Figures 3B, C**). We observed significant differences in the prognostic efficacy based on two molecular subtypes (**Figure 3D**). The IC2 subtype was correlated with a worse overall prognosis than the IC1 subtype. Using the same approach, we validated these findings in a separate cohort (GSE-LUAD) (**Figure 3E**), which suggested the potential application of the two molecular subtypes given our reproducible results across independent study cohorts.

**Figure 4:**
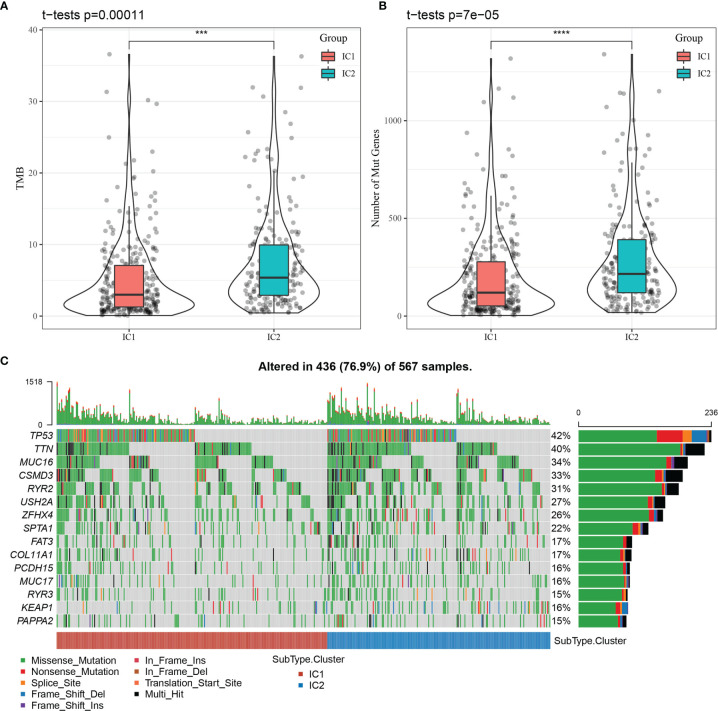
Relationship between tumor mutation burden (TMB) and molecular subtypes. **(A)** Distribution of TMB for molecular subtype samples. **(B)** Distribution of the number of mutations for molecular subtype samples. Rank sum test was used to determine the p-value, where ***p < 0.001, ****p < 0.0001. **(C)** Mutation features of significantly mutated genes in samples of each molecular subtype.

We explored the clinical features of the molecular subtypes in the TCGA-LUAD dataset, discovering 1) the surviving fraction differed significantly between the two subtypes, with higher mortality in the IC2 group; 2) the proportions of T-stage patients differed significantly between the two subtypes, with a higher proportion of T2, T3, and T4 patients in the IC2 group; and 3) the proportion of patients with advanced disease stage (II, III, and IV) was significantly higher in the IC2 group than the IC1 group (**Supplementary Figure 6**). We further analyzed differences in tumor mutation burden (TMB) between the two molecular subtypes (**Figure 4A**), determining a significant difference in TMB between the IC1 and IC2 groups. Further, the number of mutant genes also differed significantly between these molecular subtypes (**Figure 4B**). Among a total of 9780 genes screened for > 3 mutation frequencies, we obtained 936 genes. The top 15 mutation genes were characterized by mutation for the two molecular subtypes, as shown in **Figure 4C**.

### 3.3 Comparison of Immune Molecules and Functions in Molecular Subtypes

**Figure 5:**
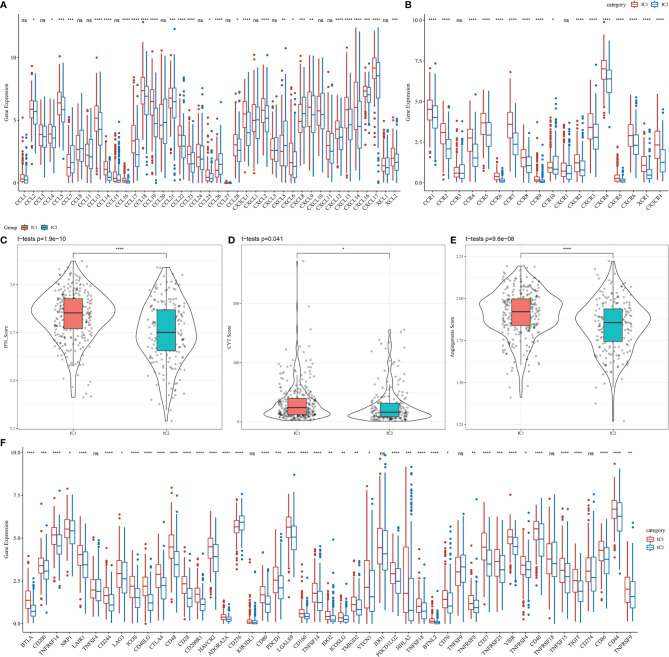
Differences in the expression of immune molecules and function between molecular subtypes in the TCGA-LUAD cohort for **(A)** chemokines, **(B)** chemokine receptors, **(C)** IFNγ, **(D)** immune T cell lysis activity, **(E)** angiogenesis scores, and **(F)** immune checkpoint genes. Significance was determined using ANOVA, where *p < 0.05; **p < 0.01, ***p < 0.001, ****p < 0.0001. ns, no significance.

We analyzed the differential expression of immune molecules in the two molecular subtypes using the TCGA-LUAD cohort. As shown in **Figure 5A**, the expression of 28 out of 41 chemokines (68.29%) differed significantly between the molecular subtypes, suggesting differing degrees of immune cell infiltration, which may lead to differences in tumor progression and immunotherapeutic effects. Furthermore, 16 out of 18 chemokine receptor genes (88.89%) were significantly differentially expressed in the molecular subtypes (**Figure 5B**). CD8^+^ T cells in the TME can produce interferon-γ (IFNγ), which stimulates the upregulation of T cell exhaustion markers over time, such as PD-1/PD-L1 and IDO1 (19, 20). Upregulation of IDO1 expression has been positively correlated with poor prognosis, tumor progression, and metastasis (21, 22). The Th1/IFNγ gene signatures were extracted as previously described (23), and IFNγ scores were calculated for each sample using the single-sample GSEA method. We found significant differences in IFNγ scores among molecular subtype samples, with higher IFNγ scores in the IC1 group than in the IC2 group (**Figure 5C**). Additionally, the intra-tumor immune T cell lysis activity of each sample was assessed using mean values of GZMA and PRF1 expression levels, as previously described (24), revealing significant differences between the two molecular subtypes (**Figure 5D**). Interestingly, the IC1 group displayed higher immune T cell lysis activity than the IC2 group. Finally, angiogenesis-associated gene sets were obtained from a previous study (25), and angiogenesis scores were calculated for each sample. The IC1 group displayed significantly higher angiogenesis scores than the IC2 group (**Figure 5E**).

Among 47 immune checkpoint-associated genes obtained from the literature (23), the expression of 41 checkpoint-associated genes (87.23%) differed significantly between the molecular subtypes, as shown in **Figure 5F**. Most of these genes were significantly more expressed in the IC1 group than in the IC2 group, suggesting that patients with different subtypes may differ in their response to immunotherapy.

**Figure 6:**
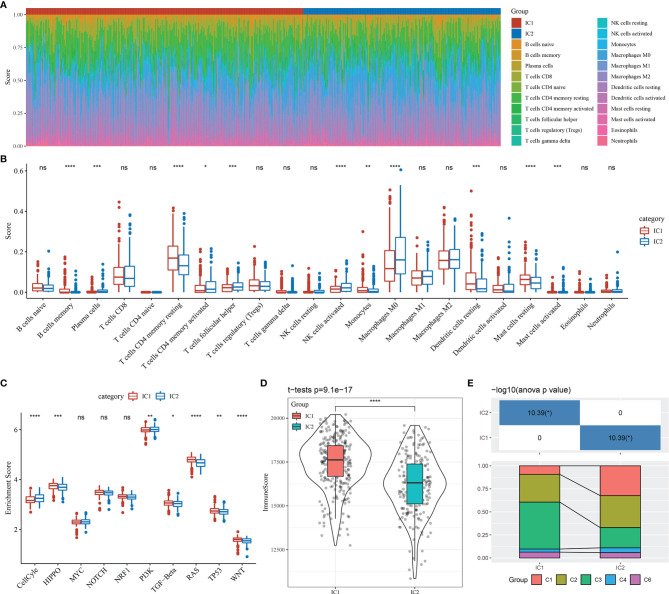
Immunological features and pathway characteristics of molecular subtypes. **(A)** Proportions of 22 immune cell types in molecular subtype samples. **(B)** Differences in immune cell scores of 22 immune cell components between molecular subtype samples. **(C)** Differences in enrichment scores of ten pathways associated with tumor abnormalities between molecular subtypes. **(D)** Distribution of immune infiltration scores between molecular subtype samples. **(E)** Comparison of the molecular subtypes with five previously identified pan-cancer immunophenotypes. *p < 0.05; **p < 0.01, ***p < 0.001, ****p < 0.0001; ns, no significance.

We subsequently identified 22 immune cell types from sample-specific immune signatures using CIBERSORT. The distribution of these immune cell types in the molecular subtype samples is shown in **Figure 6A**, and differences in immune cell score for each immune cell type between the molecular subtypes are shown in **Figure 6B**. The immune cell scores indicated significant differences in the proportion of immune cell features between the molecular subtypes, including memory B cells, CD4^+^ memory T cells, M0 macrophages, activated natural killer cells, resting dendritic and mast cells, which may play an important role in LUAD.

A prior study reported ten canonical pathways that displayed differences across multiple cancer types (26). In the current study, seven of the ten pathways differed significantly between the molecular subtypes of LUAD. Specifically, the cell cycle and PI3K pathways were mainly enriched in the IC2 subtype (**Figure 6C**).

Immune infiltration analysis indicated that the IC1 group had the highest immune microenvironment infiltration, as shown in **Figure 6D**. Further, most of the immune checkpoint-associated genes were significantly more expressed in the IC1 group than in the IC2 group, which may account for the better prognosis of patients with the IC1 subtype.

Finally, we compared our molecular subtypes with five previously identified pan-cancer immunophenotypes by utilizing molecular subtype data of these samples from the literature (26). The molecular subtypes of LUAD differed significantly from the five published immunophenotypes (**Figure 6E** and **Supplementary Figure 7**). The proportion of patients with the C1 immune subtype and poor prognosis was significantly higher in the IC2 group than in the IC1 group, while the proportion of patients with the C3 immune subtype and the better prognosis was significantly lower in the IC2 group than in the IC1 group. These findings suggested that the two molecular subtypes of LUAD complemented the five published immune subtypes.

### 3.4 Potential Clinical Impact of Molecular Subtypes

**Figure 7:**
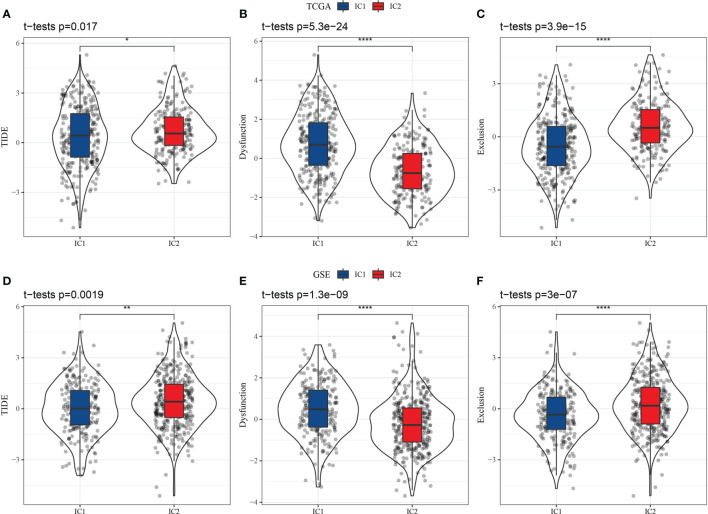
Differential analysis of T cell dysfunction and exclusion (TIDE) between molecular subtypes in two datasets (TCGA-LUAD and GSE-LUAD). **(A)** TIDE scores in TCGA-LUAD samples. **(B)** T cell dysfunction scores in TCGA-LUAD samples. **(C)** T cell rejection scores in TCGA-LUAD samples. **(D)** TIDE scores in GSE-LUAD samples. **(E)** T cell dysfunction scores in GSE-LUAD samples. **(F)** T cell rejection scores in GSE-LUAD samples. *p < 0.05; **p < 0.01, ****p < 0.0001.

Higher T cell dysfunction and exclusion (TIDE) prediction scores indicate a higher likelihood of immune escape, suggesting that patients are less likely to benefit from immunotherapy. As shown in **Figure 7A**, the TIDE scores of the IC2 group were significantly higher than those of the IC1 group in the TCGA-LUAD dataset, indicating that patients with the IC1 subtype could benefit more from immunotherapy than those with the IC2 subtype. Further, we compared the predicted T cell dysfunction and rejection scores for the molecular subtypes (**Figures 7B, C**), observing that the IC2 group had lower predicted T cell dysfunction scores and higher T cell rejection scores than the IC1 group. These findings may explain the poorer prognosis of the IC2 group and the better prognosis of the IC1 group. Moreover, these results were also observed in the GSE-LUAD dataset (**Figures 7D**–**F**).

**Figure 8:**
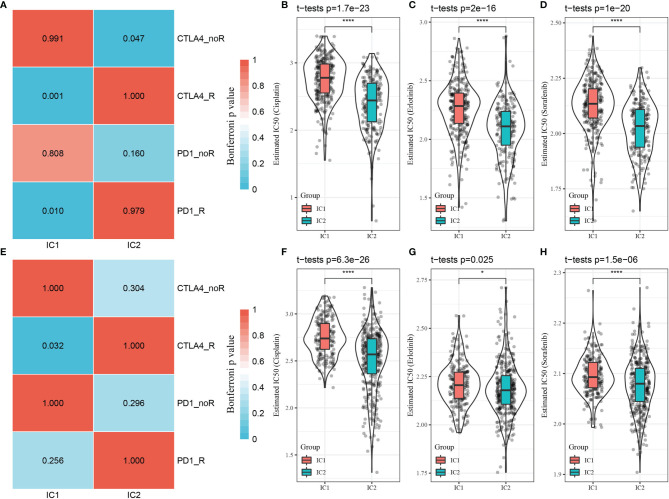
Differential analysis of immunotherapy/chemotherapy response of molecular subtypes. **(A)** TCGA-LUAD submap analysis indicating that patients with the IC1 subtype could be more sensitive to CTLA4 and PD-1 (Bonferroni-corrected p < 0.05). **(B–D)** Box plots of estimated IC50 values in the TCGA-LUAD dataset. **(E)** GSE-LUAD submap analysis indicating that patients with the IC1 subtype could be more sensitive to CTLA4 (Bonferroni-corrected p < 0.05. **(F–H)** Box plots of estimated IC50 values in the GSE-LUAD dataset. *p < 0.05; ****p < 0.0001.

To further validate whether the findings observed from the TIDE analysis applied to patient outcomes in the GSE-LUAD and TCGA-LUAD datasets, we compared patient responses to immunotherapy and chemotherapy for the two molecular subtypes in the GSE 91061 dataset using subclass mapping (submap), with lower P-values indicating higher similarity. The results indicated that the patients with the IC1 subtype in the TCGA-LUAD dataset were more sensitive to both CTLA4 and PD-1 inhibitors, but only CTLA4 monotherapy was effective for patients with the IC1 subtype in the GSE-LUAD dataset, as shown in **Figures 8A** **, E**. The responses of patients with different subtypes to conventional chemotherapeutic and targeted drugs, including cisplatin, erlotinib, and sorafenib, were also analyzed. Indeed, patients with the IC2 subtype were more sensitive to these three drugs compared to those with the IC1 subtype (**Figures 8B**–**D** **, F**–**H**).

### 3.5 Construction of the Prognostic Risk Model Based on CD8^+^ T Cell-Associated Genes

**Figure 9:**
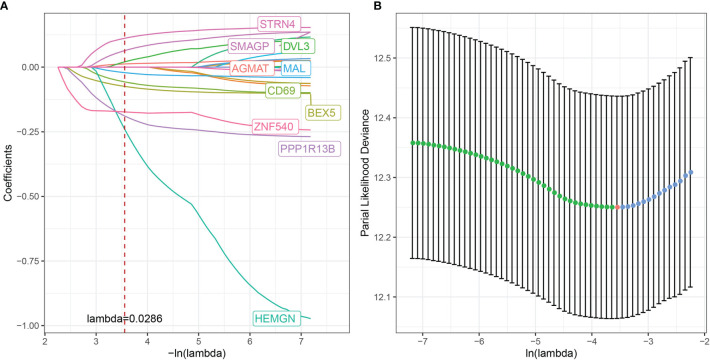
Multivariate risk analysis using the training set for construction of a prognostic risk model based on CD8^+^ T cell-associated genes. **(A)** Trajectory of each independent variable, where the horizontal axis represents the log value of the independent variable, lambda, and the vertical axis represents the coefficient of the independent variable. **(B)** Confidence interval under each lambda.

Although we identified 20 CD8^+^ T cell-associated genes that were prognostically relevant in independent datasets, prioritizing genes with the most significant clinical impact while maintaining high accuracy is necessary to simplify clinical applications for predicting therapeutic responses. Therefore, we applied Lasso Cox regression analysis to reduce the number of genes in the risk model using the *glmnet* package in R. The trajectory of each independent variable is shown in **Figure 9A**. As lambda gradually increased, the number of independent variable coefficients approaching zero also increased. Subsequently, the model was constructed using 10-fold cross-validation with the confidence intervals for each lambda shown in **Figure 9B**. The model achieved optimum performance when lambda = 0.0286. The top 10 CD8^+^ T cell-associated genes were selected as target genes for further downstream analysis, including brain-expressed X-linked 5 (BEX5), CD69, HEMGN, MAL, PPP1R13B, zinc finger 540 (ZNF540), AGMAT, disheveled protein 3 (DVL3), SMAGP, and STRN4.

These ten genes were then subjected to multivariate Cox analysis, and the risk coefficient for each gene was calculated using the RiskScore formula:


$$\begin{array}{c} RiskScore=-0.103*BEX5-0.092*CD69-0.695*HEMGN-0.042*MAL-0.279\\ {}*PPP1R13B-0.189*ZNF540+0.017*AGMAT+0.073*DVL3+0.119\\ {}*SMAGP+0.151*STRN4 \end{array}$$

**Figure 10:**
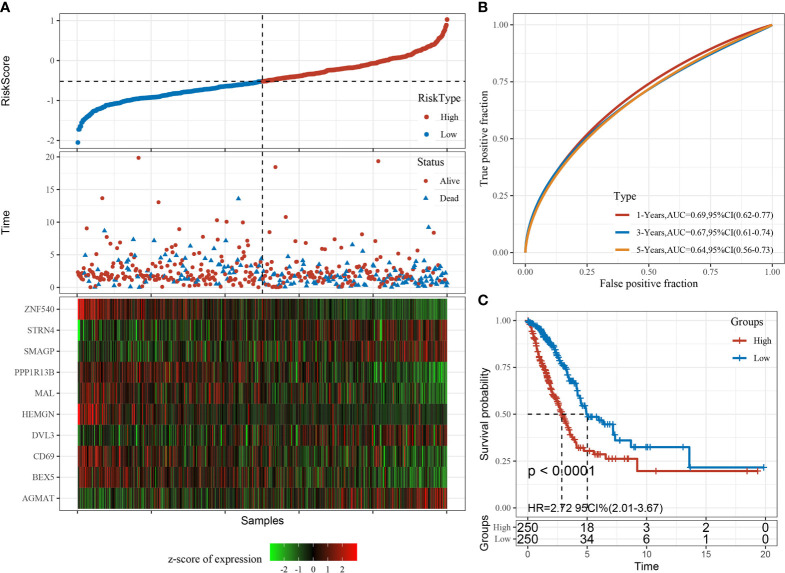
Construction and evaluation of the prognostic risk model based on CD8^+^ T cell-associated genes using the training set. **(A)** RiskScore, time to live (TTL), and survival status after applying the 10-gene signature to the TCGA-LUAD training set. **(B)** Receiver operator curves and area under the curve based on the 10-gene signature. **(C)** Kaplan–Meier survival curves for high- and low-risk groups based on the 10-gene signature using the TCGA-LUAD training set.

The RiskScore was normalized to the expression level in the sample. The distribution of RiskScore values for samples in the training dataset is shown in **Figure 10A**. Receiver operator curve analysis of the prognostic classification of RiskScore was performed using the *timeROC* package in R. The prognostic prediction efficiency of the risk model was tested at one, three, and five years, revealing high area under the curve values (**Figure 10B**). Finally, the RiskScore was used to classify high- and low-risk groups according to the median value. Kaplan–Meier curves were plotted (**Figure 10C**), displaying a significant difference between the high- and low-risk groups (P < 0.0001).

The risk model was then validated using the independent validation dataset, GSE-LUAD. The receiver operator curve analysis of the prognostic classification of RiskScore was performed as previously described, and the RiskScore was used to classify high- and low-risk groups according to the median value. Kaplan–Meier curves were plotted as shown in **Supplementary Figure 8**, displaying a significant difference between the high- and low-risk groups (P < 0.0001).

### 3.6 Relationship Between RiskScore, Clinical Features, Molecular Subtypes, and KEGG Pathways

**Figure 11:**
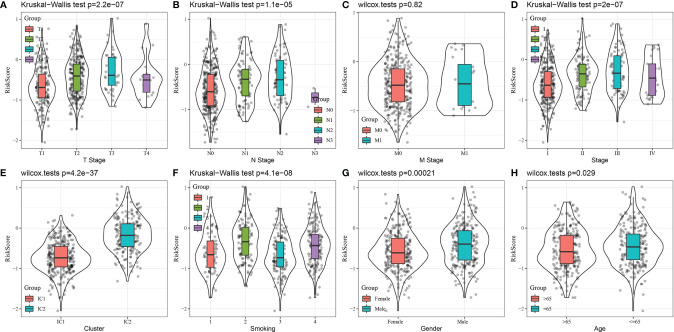
Comparison of RiskScore distribution based on clinical features and molecular subtype using the TCGA-LUAD dataset. **(A)** T stage, **(B)** N Stage, **(C)** M Stage, **(D)** Stage, **(E)** Cluster, **(F)** Smoking, **(G)** Gender, **(F)** Age.

The relationship between RiskScore, clinical features, and molecular subtypes was investigated in the TCGA-LUAD dataset. We found significant differences in RiskScore corresponding to T-stage, N stage, stage (I-IV), molecular subtype, smoking status, sex, and age (**Figure 11**, P < 0.05).

To assess the relationship between RiskScore and biological function, gene expression profiles were selected for single-sample GSEA analysis using the *GSVA* package in R, and single-sample GSEA scores were obtained for each function. The correlations between these functions and RiskScore were further calculated, and functions with correlations > 0.4 were clustered based on correlation coefficient value. Among the 19 top KEGG pathways with the most linear correlations with RiskScore, 14 and five functions were positively and negatively correlated with RiskScore, respectively. These KEGG pathways were clustered according to enrichment score, as shown in **Supplementary Figure 9**. The top KEGG pathways included the P53 signaling pathway, base excision repair, cell cycle, mismatch repair, DNA replication, and other tumor-associated pathways.

### 3.7 Analysis of the Constructed Risk Model Based on 10-Gene Signature

**Figure 12:**
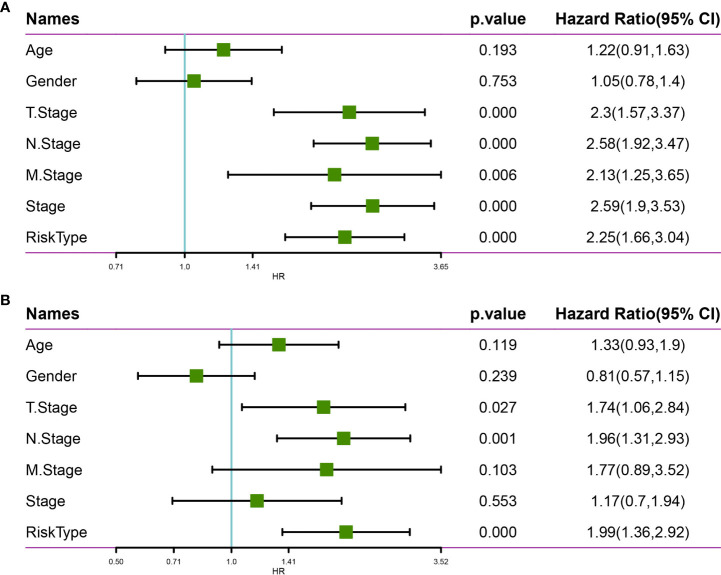
Univariate and multivariate analysis of the risk model based on the 10-gene signature using the TCGA-LUAD dataset. **(A)** Univariate Cox regression analysis. **(B)** Multivariate Cox regression analysis.

We employed univariate Cox regression analysis to test the clinical independence of the risk model based on the 10-gene signature, revealing that RiskScore was significantly correlated with survival (**Figure 12A**). In addition, multivariate Cox regression analysis demonstrated that after adjusting for biological variables, RiskScore was still significantly correlated with survival (HR = 1.99, 95% CI = 1.36-2.92, P < 1e-5) (**Figure 12B**). The above results confirmed that our risk model possessed good predictive performance for clinical applications.

**Figure 13:**
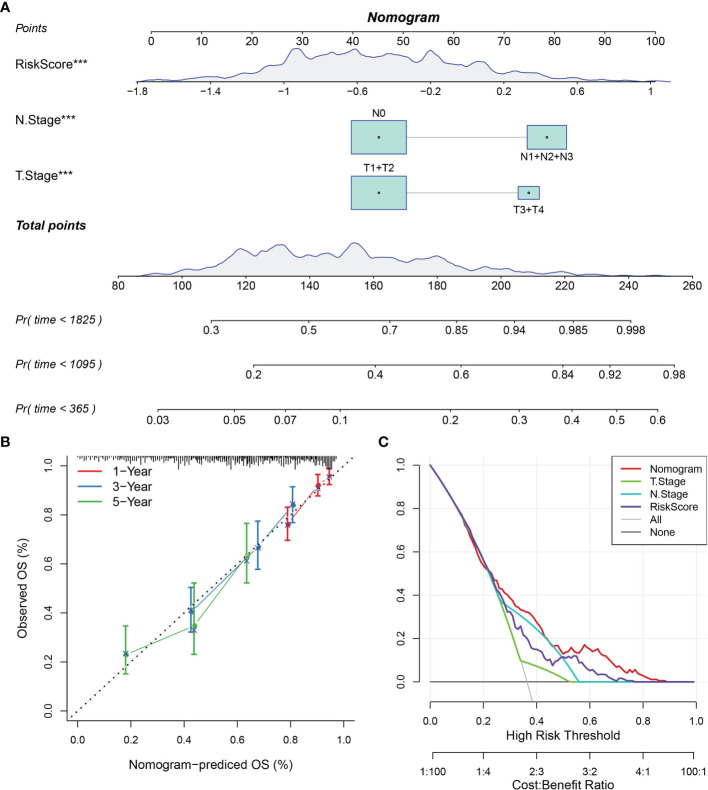
Nomogram and forest plot constructed with RiskScore and clinical features using the TCGA-LUAD dataset. **(A)** Nomogram of RiskScore, TNM stage, and recurrence. **(B)** Correction plot of the nomogram. **(C)** Decision curve analysis plot.

A nomogram was constructed using stage and recurrence data and combined with RiskScore using the full TCGA-LUAD dataset (**Figure 13A**) to enable visualization of the risk model in an intuitive, practical, and efficient way. The RiskScore feature had the greatest impact on predicting survival outcomes, indicating that the 10-gene-based risk model could outperform current methods of determining disease prognosis. The correction curve suggested that our model was highly accurate (**Figure 13B**). Further, the decision curve analysis plot of T-stage, N stage, RiskScore, and nomogram values indicated that the nomogram had better predictive performance than other methods (**Figure 13C**).

### 3.8 Prediction Efficacy of the Constructed Risk Model for Immunotherapy

**Figure 14:**
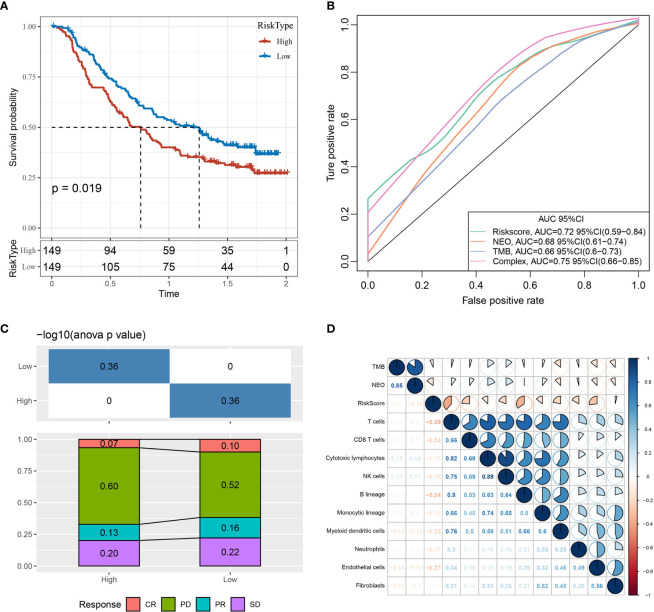
Prediction efficacy of the risk model based on the 10-gene signature. **(A)** Kaplan–Meier curves of high- and low-risk groups using the Imvigor210 dataset. **(B)** Evaluation of the risk model against standard prediction models of immunotherapy response using the Imvigor210 dataset. **(C)** Corresponding stacked plots of immunotherapy response among high- and low-risk groups in the Imvigor210 dataset. **(D)** Correlation between RiskScore, immune score, TMB, and NEO using the Imvigor210 dataset. CR, complete response; PR, partial response; SD, stable disease; PD, progressive disease.

An immunotherapy dataset (Imvigor210) containing transcriptomic data was retrieved to explore whether the constructed risk model based on the 10-gene signature could predict the efficacy of immunotherapy. The Imvigor210 dataset included expression data of human metastatic urothelial carcinoma samples from patients who responded or did not respond to anti-PD-L1 immunotherapy. The Kaplan–Meier curves demonstrated that patients with higher RiskScore values were associated with poorer survival outcomes following immunotherapy treatment (**Figure 14A**).

Furthermore, the risk model had higher area under the curve values compared to standard prediction models of immunotherapy response (**Figure 14B**). There was no significant difference between immunotherapy response and non-response in the high- and low-risk groups (**Figure 14C**). The RiskScore, TMB, NEO, and immune cell scores of the Imvigor210 samples were calculated and correlated using the *MCPcounter* package in R, demonstrating that RiskScore was negatively correlated with TMB and NEO and weakly correlated with immune cell score (**Figure 14D**).

**Figure 15:**
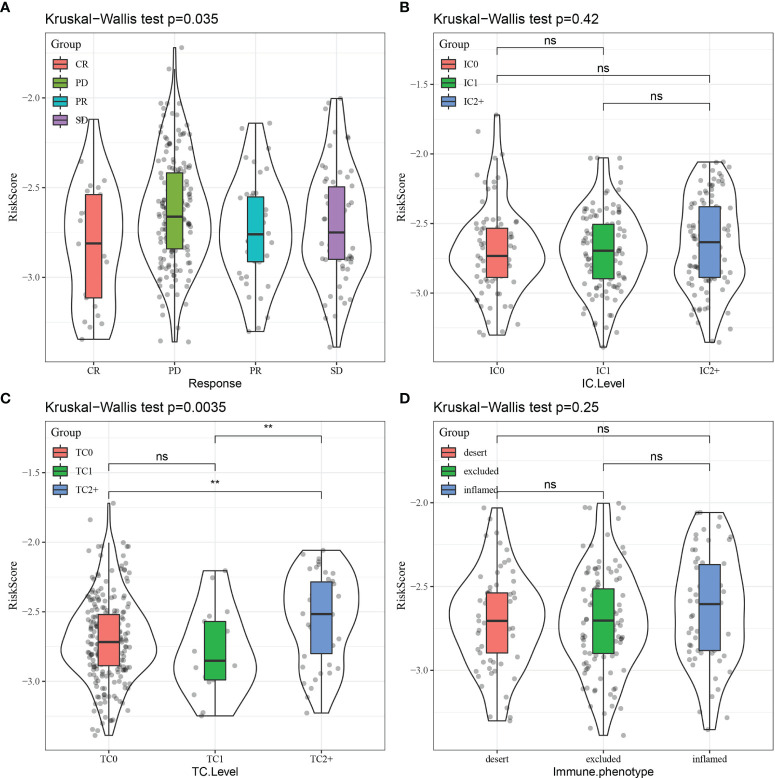
Comparison of RiskScore distribution across different subgroups for **(A)** immunotherapy response, **(B)** immune cell (IC) level, **(C)** tumor cell (TC) level, and **(D)** immune phenotype. CR, complete response; PR, partial response; SD, stable disease; PD, progressive disease. **p < 0.01; ns, no significance.

Finally, RiskScore was compared across different groups, revealing that different immunotherapy response (**Figure 15A**) and tumor cell level (**Figure 15C**) groups displayed significant differences in RiskScore, while a trend toward a difference in RiskScore was observed in different immune cell levels (**Figure 15B**) and immune phenotype (**Figure 15D**) groups.

## 4 Discussion

In this study, we utilized the expression profile data of 14 immune cell types obtained from 13 publicly available datasets to identify 252 CD8+ T cell-associated genes by weighted correlation network analysis. To validate the accuracy of our initial screening, we employed Gene Ontology and KEGG enrichment analyses to confirm that the identified genes were closely related to immune function and tumor-related pathways. In addition, We obtained the original data of single-cell data GSE148071 (18 patient samples of lung adenocarcinoma) and performed cell annotation analysis. The results indicated that the 20 genes were not uniquely expressed in CD8+ T cells. Single-cell sequencing annotates cell subpopulations based on expression of marker genes. Some cells expressing multiple cell marker genes at the same time will lead to certain false negative in cell annotation. This might be the reason for the inconsistency between Single-cell sequencing and our results. To ensure the accuracy and reliability of the data, we rigorously validated our model predictions with multiple independent analyses. Patients in the IC2 group had a poor prognosis and advanced TNM stage compared to those in the IC1 group. Furthermore, the TMB was significantly higher in the IC2 group than in the IC1 group. Levels of chemokines and immune cell infiltration, as well as expression of angiogenesis-associated genes and immune-related pathways, differed significantly between the IC1 and IC2 groups. Moreover, the IC1 group had higher immune scores and immune checkpoint-associated gene expression than the IC2 group, which could explain the better prognostic outcome of patients in this group. Finally, the IC2 group had higher TIDE scores, indicating a higher likelihood of immune escape and thus a lower probability of benefiting from immunotherapy. Thus, differences in response to chemotherapy and targeted therapies (standard first-line therapies for LUAD) were assessed between the two groups, revealing that patients with the IC2 subtype were more sensitive to cisplatin, erlotinib, and sorafenib than those with the IC1 subtype. These results suggest that the two molecular subtypes of LUAD based on CD8^+^ T cell-associated genes can distinguish between high- and low-risk LUAD patients and potentially have a meaningful clinical impact.

Another important result of this study was the identification of a robust 10-gene signature based on CD8^+^ T cell-associated genes in LUAD patients that includes *BEX5*, *CD69*, *HEMGN*, *MAL*, *PPP1R13B*, *ZNF540*, *AGMAT*, *DVL3*, *SMAGP*, and *STRN4.* Expression of the novel 10-gene signature corresponded with predictive value in both the TCGA-LUAD and GSE-LUAD datasets and was an independent predictor of prognosis in LUAD patients. *BEX5*, a member of the BEX family, is involved in various biological functions related to normal and tumor tissues (27). A previous study demonstrated that *BEX5* mRNA levels are downregulated in LUAD tissues and have prognostic value (28). CD69, a C-type lectin receptor family member, is the earliest surface antigen expressed after T lymphocyte activation and acts as a co-stimulatory signal to promote further activation (29, 30). Compared to normal lung tissues, CD69 is less expressed in LUAD tissues and is positively associated with the apoptosis of LUAD cells (31). High expression of HEMGN, a nucleoprotein involved in regulating the proliferation and differentiation of hematopoietic cells (32), reportedly indicates a better prognosis in acute myeloid leukemia (33). Expression of *MAL*, which occurs at intermediate- to late-stage T cell differentiation, is downregulated or absent in a variety of tumor tissues, such as gastric cancer (34) and LUAD (35) and corresponds with advanced tumor progression. *PPP1R13B*, also known as apoptosis-stimulating proteins of the p53 (*ASPP1*), is a member of the newly discovered ASPP family that exerts its cancer-inhibiting effects mainly by promoting p53-mediated apoptosis (36) and is downregulated in the lung (37), kidney (38), and colon (39) cancers. ZNF540 is a newly identified member of the ZNF protein family that binds to major vault protein to inhibit the ERK signaling pathway (40). A previous study reported that high *AGMAT* expression is closely associated with poor prognosis in LUAD patients and promotes tumorigenesis through the NO-MAPK-PI3K/Akt pathway (41). DVL3 belongs to the DVL family, which is the cytoplasmic mediator of the Wnt/b-catenin signaling pathway and plays an important role in embryonic development, cell differentiation, and tumor formation (42). High DVL3 expression is associated with poor prognosis in patients with lung cancer (43, 44). *SMAGP* encodes a transmembrane glycoprotein that plays an important role in tumor invasion and metastasis (45–47). *STRN4* belongs to the striatin family and functions as a co-factor (48), particularly in cancer-related pathways (49) in prostate (50), lung (51), and gastric (52) cancers. In the current study, *ZNF540* and *SMAGP* were identified as prognostic markers for LUAD for the first time, but their specific roles and potential regulatory mechanisms require further investigation.

The novel 10-gene signature developed in the current study was closely associated with the p53-signaling pathway. p53 regulates the expression of a wide variety of genes, whose functions include promoting apoptosis and inhibiting growth, cell cycle progression, DNA repair, genotoxicity, and senescence following cellular stress. Several additional enriched pathways were correlated with RiskScore, including base excision repair, mismatch repair, and DNA replication, likely mediated through p53. Overall, the p53 pathway likely plays an essential role in the progression of LUAD.

Traditional antitumor treatments, such as chemotherapy, can achieve certain efficacy, but the overall survival of patients with LUAD is compromised by several factors, including normal tissue toxicity and multi-drug resistance (3). In recent years, targeted tumor therapies such as gefitinib and crizotinib have been effective against specific tumors, but their application is limited to a fraction of LUAD patients, and drug resistance eventually develops (53). The use of immune checkpoint blockade (ICB) antibodies has achieved significant efficacy in lung cancer (54), but only 30% of lung cancer patients display long-term benefits (55). Therefore, biomarkers that can effectively predict the efficacy of ICB therapy are urgently needed. TIDE score, which corresponds to T cell dysfunction, has been shown to effectively predict the efficacy of ICB treatment (56). In the current study, our constructed risk model demonstrated better performance than standard prediction models in predicting the survival of metastatic urothelial carcinoma patients receiving immunotherapy, demonstrating the model’s clinical potential for evaluating candidate LUAD patients to receive ICB therapy.

Although our risk model based on the 10-gene signature robustly determined LUAD prognosis following immunotherapy, our study has several limitations. First, this was a retrospective study. Thus, validation with prospective samples is required. Second, only the prognostic value and clinical significance of the 10-gene signature were analyzed; therefore, the mechanisms underlying gene expression and CD8^+^ T cell activity warrant further exploration.

In conclusion, this study identified two robust molecular subtypes of LUAD based on CD8^+^ T cell-associated genes, with each subtype demonstrating significantly different patient prognoses, clinical characteristics, immune cell phenotypes, and therapeutic responses. In addition, the constructed risk model based on the 10-gene signature performed better than current methods in predicting the prognosis and immunotherapeutic response of LUAD patients.

## Data Availability Statement

Publicly available datasets were analyzed in this study and can be found here: GSE13906, GSE23371, 650 GSE27291, GSE27838, GSE28490, GSE28726, GSE37750, GSE39889, GSE42058, GSE49910, 651 GSE59327, GSE6863, GSE8059, GSE37745, GSE19188, GSE50081, GSE30219, GSE31210, and https://www.cancer.gov/tcga.

## Author Contributions

HP conceived, designed, planned the study, and interpreted the results. MZ and JM analyzed the data. QG and SD acquired the data. MZ and JM drafted the manuscript. All authors contributed to the article and approved the submitted version.

## Funding

This research was funded by the Natural Science Foundation of Heilongjiang Province (Grant No. LH2019H040), the China Postdoctoral Science Foundation (Grant No. 2018M640309), the Heilongjiang Province Postdoctoral Science Foundation (Grant No. 2018JTC), and Haiyan Foundation of Harbin Medical University Cancer Hospital (Grant No. JJMS2021-14).

## Conflict of Interest

The authors declare that the research was conducted in the absence of any commercial or financial relationships that could be construed as a potential conflict of interest.

## Publisher’s Note

All claims expressed in this article are solely those of the authors and do not necessarily represent those of their affiliated organizations, or those of the publisher, the editors and the reviewers. Any product that may be evaluated in this article, or claim that may be made by its manufacturer, is not guaranteed or endorsed by the publisher.

## Abbreviations

LUAD, lung adenocarcinoma; TME, tumor microenvironment; TMB, tumor mutation burden; PD-1, programmed death protein 1; CTLA4, cytotoxic T lymphocyte-associated protein 4; KEGG, Kyoto Encyclopedia of Genes and Genomes; CDF, cumulative distribution function; ICs, immune clusters; IFNγ, interferon-γ; BEX, brain-expressed X-linked; ZNF, zinc finger; ASPP, apoptosis-stimulating proteins of the p53; DVL, disheveled protein; ICB, immune checkpoint blockade; TIDE, T cell dysfunction and exclusion.
