# Supplementary material for: CD8+ T Cell-Associated Gene Signature Correlates With Prognosis Risk and Immunotherapy Response in Patients With Lung Adenocarcinoma
Source: Front Immunol. 2022 Feb 22;13:806877. doi: 10.3389/fimmu.2022.806877 (PMC8902308; doi:10.3389/fimmu.2022.806877)
Supplement: Supplementary file 1 [file DataSheet_1.pdf]

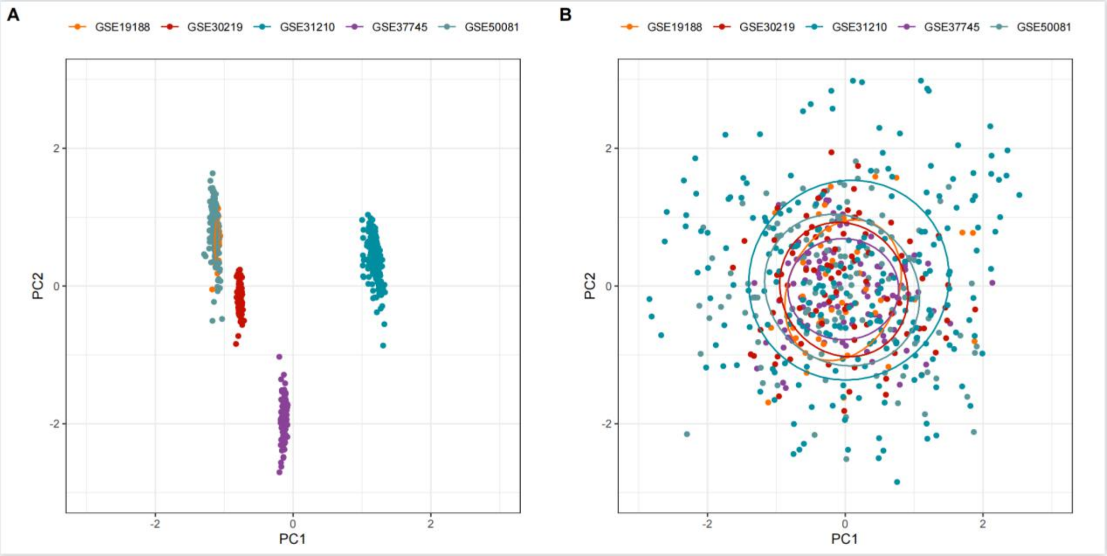

Supplementary Figure 1. Principal component analysis of the GSE-LUAD datasets (A) before and (B) after batch effect correction.

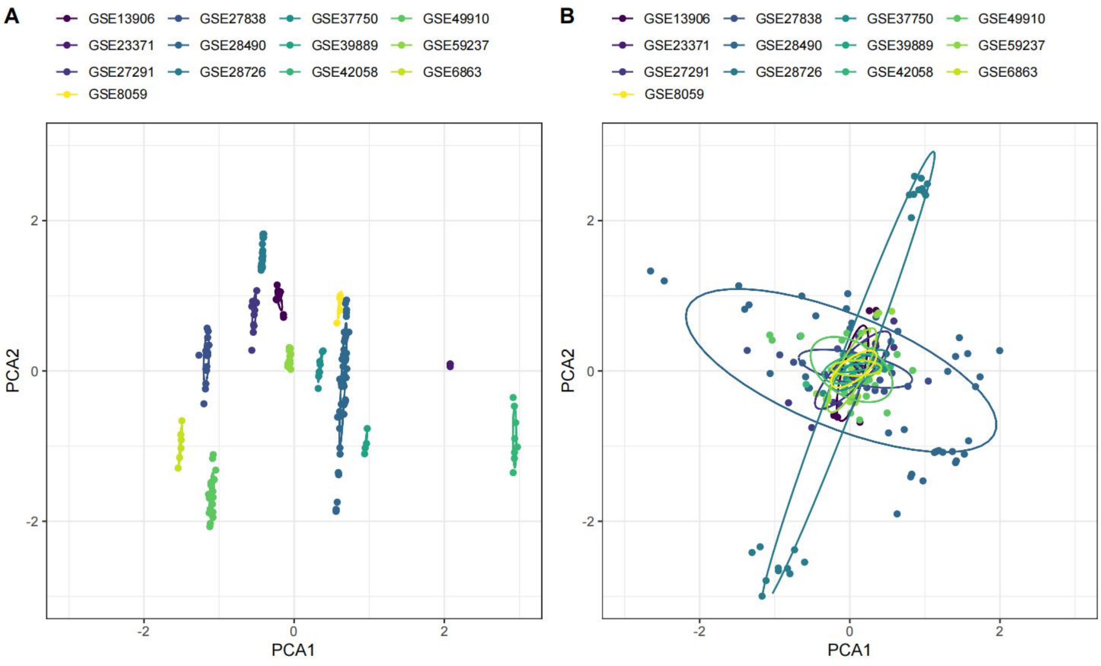

Supplementary Figure 2. Principal component analysis of the 13 immune cell-line associated datasets (A) before and (B) after batch effect correction.

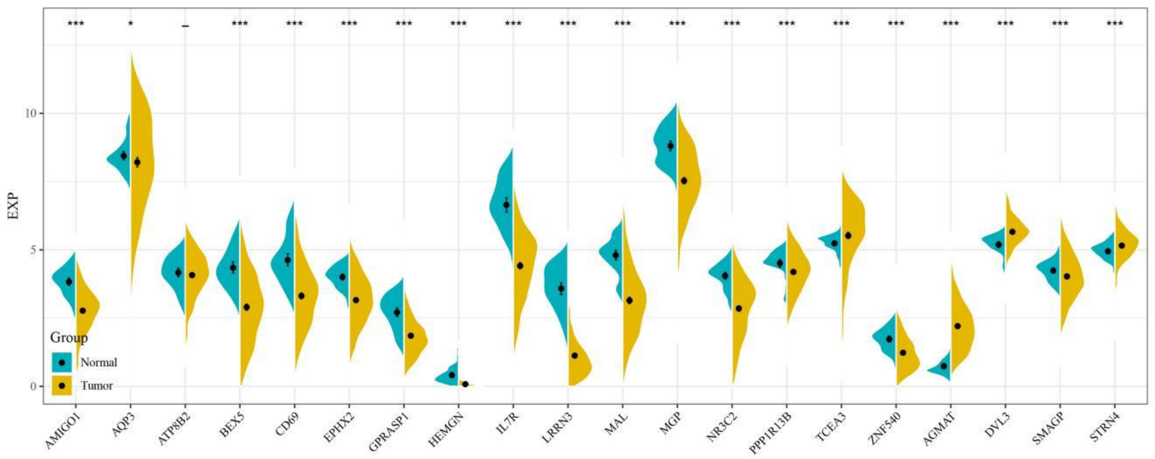

Supplementary Figure 3. Differential 20 expression between tumor and normal tissues

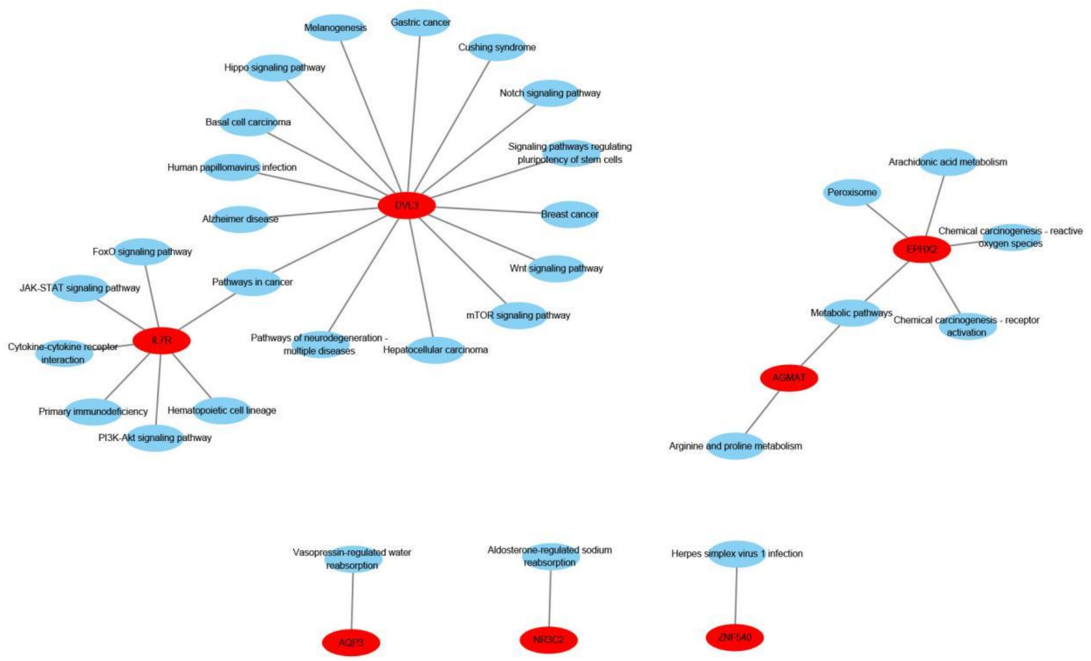

Supplementary Figure 4. PPI network and function analysis of hub gene

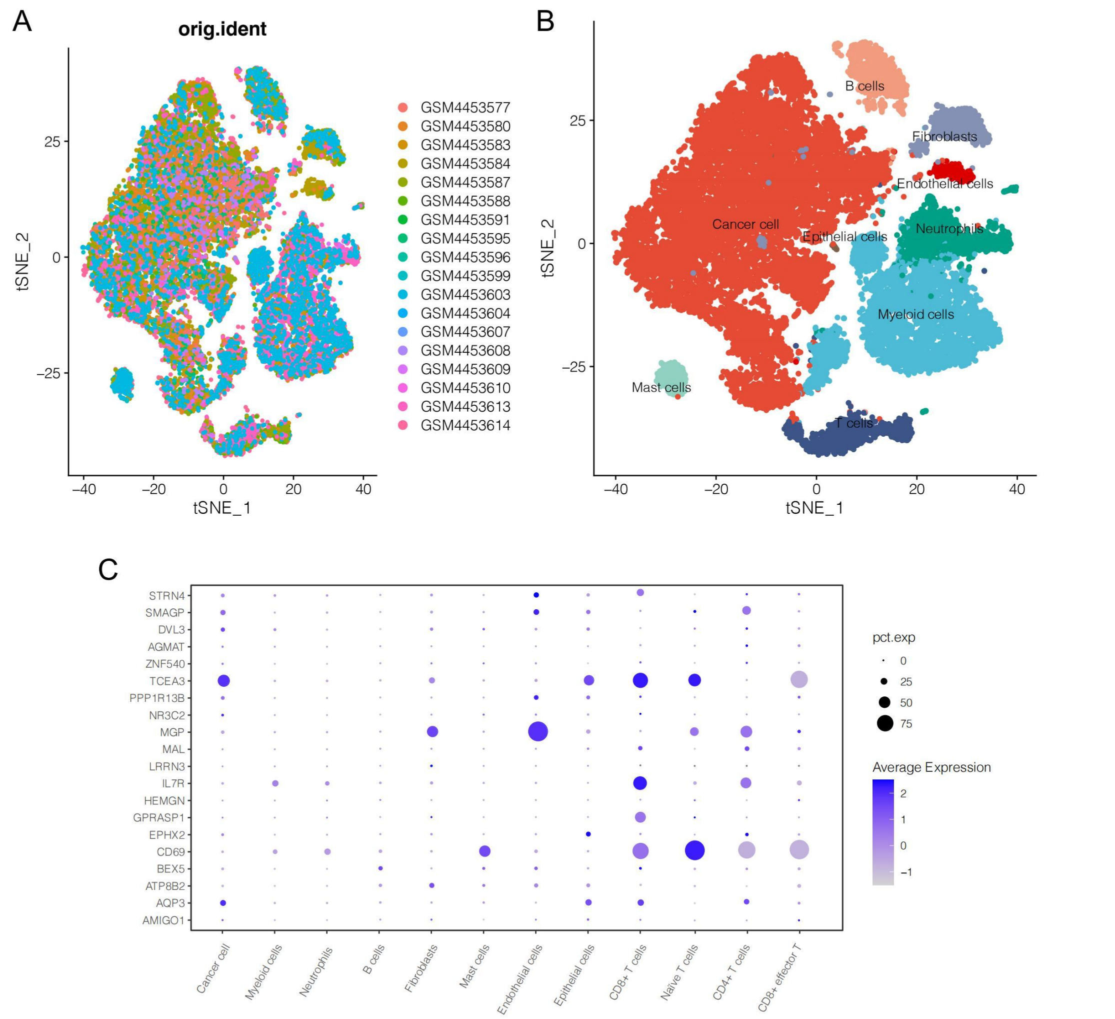

Supplementary Figure 5. (A) represents 18 samples after removing the batch effect, (B) represents cell subgroup classification. We extracted the cell data defined as T cells, and then further subdivided the clustering, using the markers provided in the literature, to subdivide the T cells into CD8+ T cells, Naïve T cells, CD4+ T cells, CD8+ effector T cells, and then plotting the expression of our 20 genes between them. (C) showed that most genes are significantly expressed in CD8+ T cells.

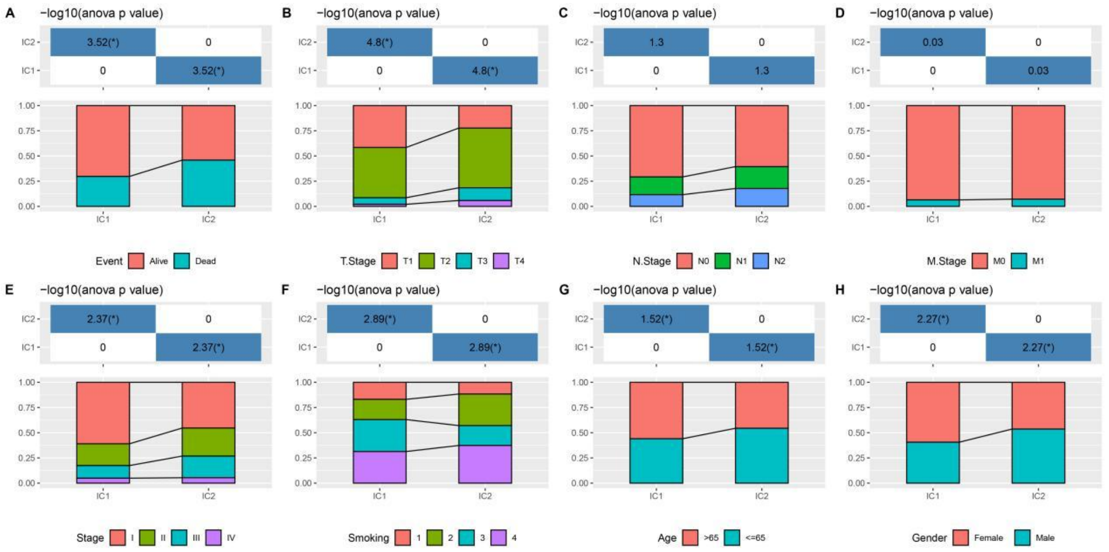

Supplementary Figure 6. Comparison of different clinical features between the two molecular subtypes in the TCGA-LUAD dataset.

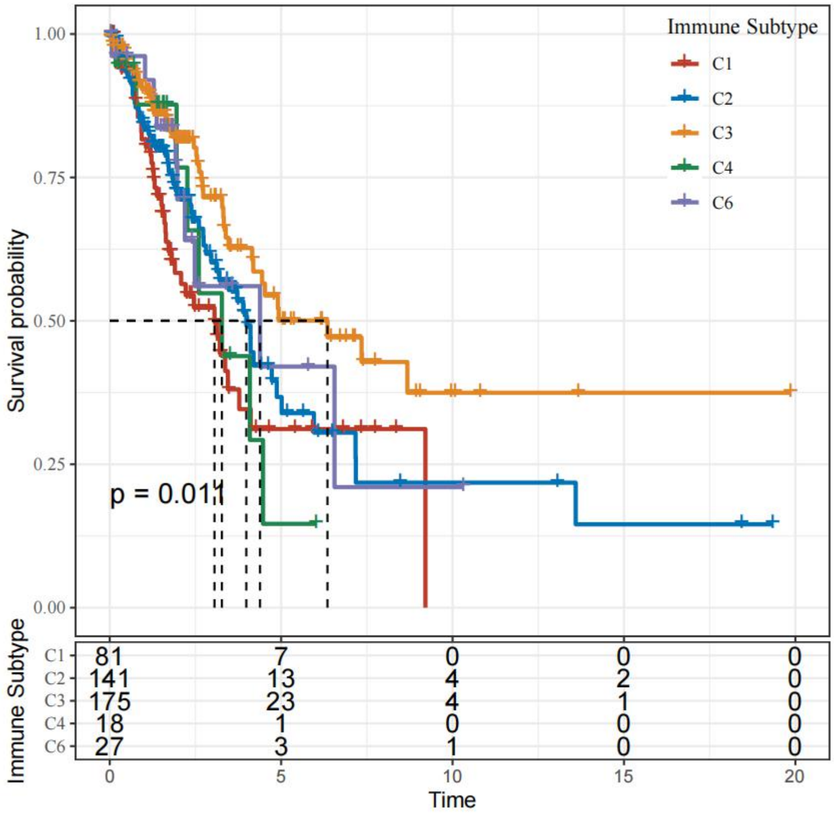

Supplementary Figure 7. Prognostic survival curve between different immune subtypes

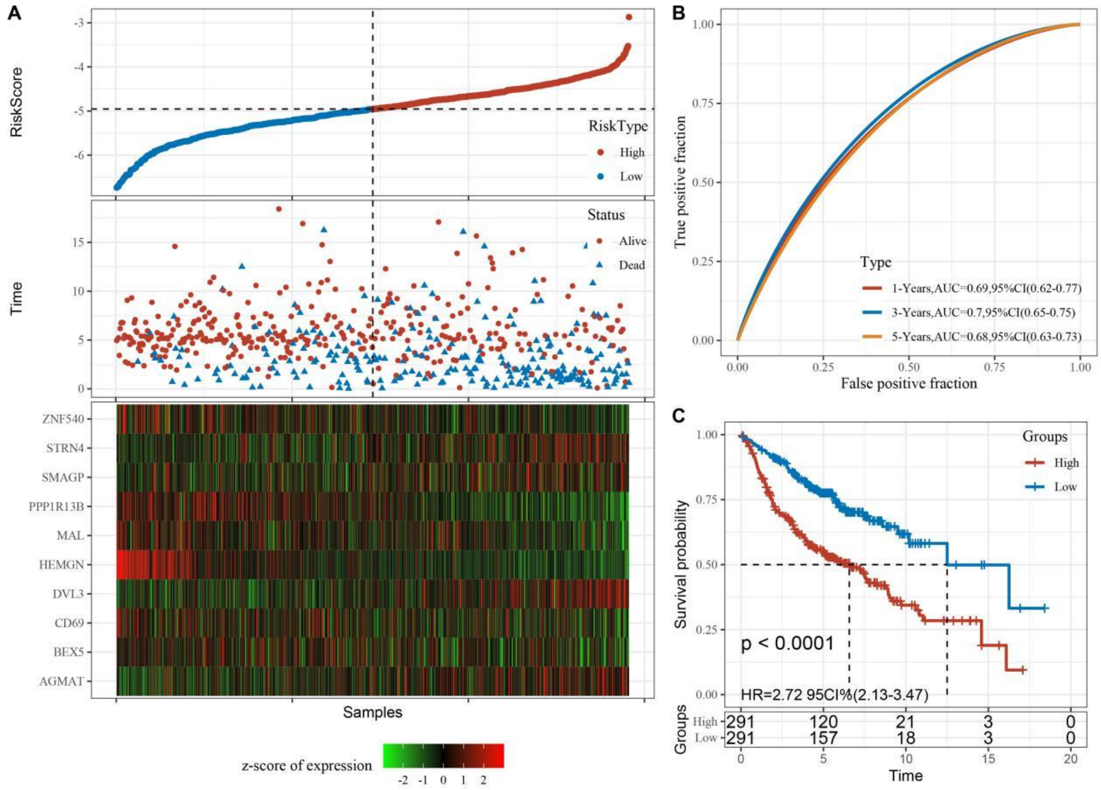

Supplementary Figure 8. Evaluation of the prognostic risk model based on CD8+ T cell-associated genes using the validation set. (A) Risk score, time to live (TTL), survival status, and 10-gene expression in the independent validation dataset, GSE-LUAD. (B) Receiver operator curves and area under the curve based on the 10-gene signature. (C) Kaplan – Meier survival curves for high- and low-risk groups based on the 10-gene signature using the independent validation dataset, GSE-LUAD.

A

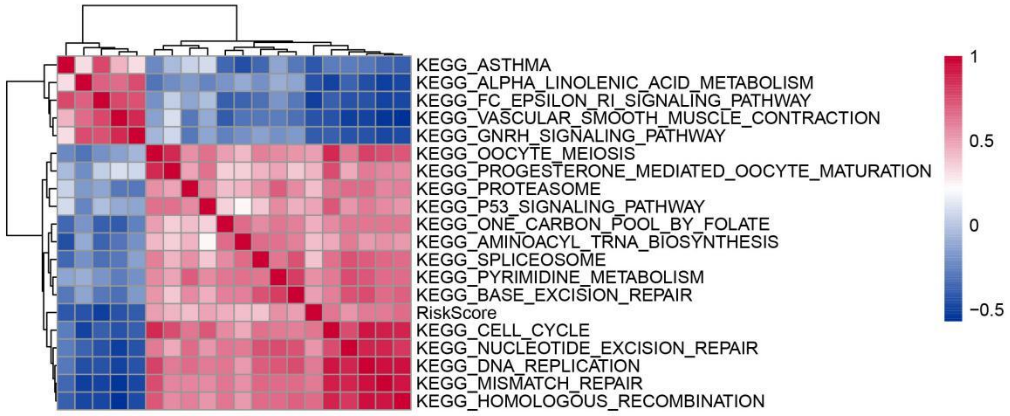

B

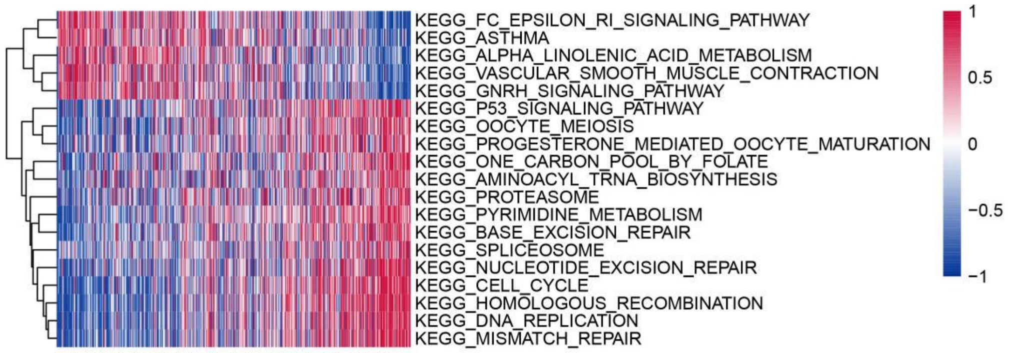

Supplementary Figure 9. Relationship between RiskScore and Kyoto encyclopedia of genes and genomes (KEGG) pathways. (A) Clustering of correlation coefficient values for KEGG pathways with a correlation > 0.4. with RiskScore. (B) Clustering of enrichment scores for KEGG pathways with a correlation > 0.4 with ssGSEA scores. The horizontal axis represents samples, with increasing RiskScores from left to right.
